# Navigating barriers to real-world evidence utilization for drug regulatory affairs and market access in Saudi Arabia

**DOI:** 10.3389/fphar.2025.1712147

**Published:** 2025-12-02

**Authors:** Hatouf H. Sukkarieh, Nourah Al Fayez, Hussain Abdulrahman Al-Omar, Nura O. Azmi, Bushra Syed, Raed A. Albar, Nawaf Al Bali

**Affiliations:** 1 Department of Pharmacology, College of Medicine, Alfaisal University, Riyadh, Saudi Arabia; 2 Department of Management, College of Business, Alfaisal University, Riyadh, Saudi Arabia; 3 Department of Clinical Pharmacy, College of Pharmacy, King Saud University, Riyadh, Saudi Arabia; 4 College of Medicine, Alfaisal University, Riyadh, Saudi Arabia; 5 Medical Education, College of Medicine, Alfaisal University, Riyadh, Saudi Arabia; 6 Executive Department of Policy and Compliance, Council of Health Insurance, Riyadh, Saudi Arabia; 7 Department of Public Health, Epidemiology, and Biostatistics, College of Medicine, Alfaisal University, Riyadh, Saudi Arabia

**Keywords:** real-world evidence, policy, regulatory affairs, reimbursement, barriers, Saudi Arabia

## Abstract

**Background:**

Data-driven, evidence-based decision-making is crucial for guiding complex and often conflicting decision-making in health systems. This study explored the barriers and challenges faced by stakeholders in generating and using real-world evidence (RWE) in Saudi Arabia, focusing on pharmaceutical regulatory, reimbursements, and research landscapes.

**Methods:**

Semi-structured interviews were conducted with Saudi stakeholders working in regulatory organizations, research institutions, and the pharmaceutical industry using a topic guide. Purposeful sampling was used to select study participants. The interview topic guide included three main open-ended questions focused on the participants’ current experiences, existing barriers, and potential interventions for overcoming challenges. Thematic analysis was used to identify barrier- and challenge-related themes and subthemes.

**Findings:**

11 stakeholders working in regulatory organizations, research institutions, and the pharmaceutical industry across Saudi Arabia were interviewed. Based on the interviews, we identified four major themes pertaining to the barriers and challenges related to advancing RWE use in Saudi Arabia: data management, local capabilities, resources, and governance. Each theme contained key subthemes.

**Conclusion:**

This study reveals critical barriers to RWE generation and utilization in Saudi Arabia. Having clear governance, policies, and guidelines for the collection, standardization, sharing, and utilization of real-world data is crucial for overcoming these barriers and challenges. A multifaceted policy approach characterized by stakeholder engagement, collaboration, and coordinated implementation efforts could help build a robust, unified RWE system that aligns with the Saudi Vision 2030 objectives for a sustainable, data-driven healthcare ecosystem.

## 1 Introduction

In today’s data-driven world, rapid advancements in health-information technology have generated an unprecedented amount of biomedical data. This surge in data availability has sparked significant interest from stakeholders across the healthcare sector, including clinicians, researchers, and policymakers (Rachmania and Basri, 2013). In healthcare, real-world evidence (RWE) refers to clinical evidence generated through the utilization of real-world data (RWD) related to patients and systems. These data include information collected from sources such as electronic health records (EHRs), insurance claims, patient-reported outcomes, and data from registries, wearable devices, and social media. The United States Food and Drug Administration defines RWE as evidence from the analysis of RWD used to inform healthcare decision-making (U. S. Food Drug Administration, 2018). The European Medicines Agency views RWE as data reflecting how treatments are performed in routine clinical practice, whereas the International Society for Pharmacoeconomics and Outcomes Research emphasizes RWE’s role in evaluating the effectiveness and value of interventions in real-life settings (Makady et al., 2017). RWE is generated by gathering data from diverse real-world clinical sources. It provides valuable and supplementary insights from none randomized controlled trials (RCTs) studies that could potentially enhance healthcare decision-making beyond what RCTs can offer (Berger et al., 2017; Dang, 2023).

RCTs are widely recognized as the gold standard for evidence-based medicine; however, they often fail to reflect real-world patient conditions, thereby limiting their generalizability and external validity (Peng et al., 2022). This limitation highlights the need for RWE, which is generated during routine clinical practice and on other relevant platforms (Azoulay, 2022; Peng et al., 2022). For instance, in cases where patients are ineligible for RCTs, such as oncology, RWE plays a particularly important role in evaluating the long-term health outcomes and effectiveness of therapies. It can capture how therapies perform across diverse populations, particularly for those excluded from RCTs, identifying rare or delayed adverse events, informing regulatory and policy decisions, and supporting post-marketing surveillance. Additionally, RWE helps optimize patient care and demonstrates cost-effectiveness while estimating disease burden and epidemiology, treatment utilization, and compliance; evaluating medication safety; and guiding strategic healthcare investment (Mahajan, 2015; Corrigan-Curay et al., 2018).

Unlike RCTs, RWE studies often feature larger sample sizes, enabling quicker and more insightful analytics and facilitating subgroup analyses to improve the generalizability of the results across diverse patient populations (Sherman et al., 2016; Kim et al., 2018; Dang, 2023). However, RWE studies have certain limitations. These include potential biases owing to nonrandomized data sources and variability in data quality and completeness, which may limit the reliability of the findings (Huang et al., 2016; Sherman et al., 2016). By combining RCTs and RWE, insights into treatment outcomes across varied clinical scenarios and patient demographics can be enriched, allowing for better decision-making at all levels (Peng et al., 2022).

RWE is increasingly recognized as a valuable complement to RCTs, particularly in the context of medication approval and reimbursement decisions (Al-Omar et al., 2021). Recent studies have shown that RWE can support regulatory evaluations by providing insights into a drug’s performance in routine clinical settings and addressing gaps in efficacy, safety, and usage across broader populations. Additionally, RWE informs health technology assessment and payer decisions by incorporating patient-centered outcomes and supporting value-based pricing models (Ramos-Goni et al., 2017; Risor et al., 2017).

In Saudi Arabia, generating RWE faces challenges, such as the absence of national-level health data standards, limited access to high-quality datasets, and the lack of a cohesive regulatory framework to support systematic data collection and utilization (Alzarea et al., 2022). Despite these challenges, there are significant opportunities in Saudi Arabia to enhance the capacity to generate and leverage RWE to advance patient-centered care while transformative changes in healthcare are underway. Addressing these key challenges will help ensure the generation of a reliable, robust, accessible, and secure RWE (Farghaly et al., 2023). Against this background, this study aimed to gain an in-depth understanding of the barriers and challenges facing health system stakeholders in generating and using RWE in the domain of pharmaceutical regulatory and reimbursement in Saudi Arabia and to interpret these phenomena from their perspectives.

## 2 Methods

### 2.1 Qualitative study design

A descriptive qualitative research approach was employed to describe the landscape of RWE and RWD generation in Saudi Arabia. Purposeful sampling was used to interview participants with extensive experience and leadership roles relevant to the use of RWE in the field of pharmacy regulatory and reimbursement roles in Saudi Arabia. The number of participants was determined based on the principle of data saturation, a standard approach in qualitative research.

### 2.2 Participant recruitment

Potential participants were identified using a two-stage process. First, the research team compiled a list of key organizations. Second, senior stakeholders in these organizations who met the inclusion criteria were identified through professional networks, institutional websites, and nominations from initial contacts. The inclusion criteria were senior stakeholders from diverse sectors (governmental, semi-governmental, and private), including regulatory bodies, academia, research institutions, local and multinational pharmaceutical companies, healthcare data and consulting firms, and professional societies. Variation among eligible participants was maximized based on their work and years of experience to ensure broad perspectives across sectors. All participants were based in Saudi Arabia and were directly involved in or had oversight over RWE-related activities.

### 2.3 Data collection

This study used in-depth, semi-structured interviews for data collection. The interview method was selected because of its compatibility with the research objective and its ability to generate rich, meaningful insights. The use of real-life accounts enhanced the credibility of the findings, offering a more comprehensive exploration of the participants’ experiences. An interview topic guide was used to ensure consistency among participants (Supplementary Appendix S1). It consisted of three main open-ended questions with probes and prompts to elaborate on participants’ responses; these were fully developed, reviewed, validated, modified, approved, and piloted before conducting the interviews (Supplementary Appendix S2). The interview questions addressed participants’ experiences with generating RWE, their opinions on the barriers and challenges faced by stakeholders (including themselves) regarding generating RWE, and potential interventions they could use to improve the use and maturity of RWE in Saudi Arabia.

The semi-structured interviews were conducted by H.S. and N.A. online using Zoom. They focused on gathering insights and views about the current RWE landscape, specific barriers and limitations faced by stakeholders, and potential solutions in Saudi Arabia. Supplementary Appendix S1 presents the interview guidelines. All interviews were recorded with the participants’ consent and transcribed verbatim. Transcription was performed independently by two trained individuals to enhance accuracy and ensure fidelity to the original recordings. The transcripts were then cross-checked and reviewed for consistency and completeness by the research team, ensuring that any discrepancies were resolved and that the final transcripts accurately reflected the participants’ responses.

To enhance the study’s credibility, the researchers provided a detailed description of the context and the participants’ backgrounds. Data analysis was carefully documented to ensure that the conclusions were grounded in the actual data, thus minimizing the risk of researcher bias (Moser and Korstjens, 2018). All interviews were transcribed verbatim and processed using Quirkos™ software and then reviewed by two research team members. The transcripts were then analyzed thematically using Braun and Clarke’s six-step approach (Braun and Clarke, 2006). These analyses were performed independently by N.A. and H.A., and the findings were discussed to determine the final themes and subthemes. Table 1 presents the themes and subthemes emerging from the qualitative analyses. To maintain the richness and context of participants’ experiences, direct quotes are included in the Findings section to exemplify and support each major theme and subtheme in results section.

**TABLE 1:** List of emerging themes and subthemes.

Theme	Sub-theme
Data management	Data sharing issues Data quality issues
Capabilities building	Institutionalized training and development Research and collaboration support system
Resources utilization	Raising awareness Investment in RWE
Governance	Regulators support Regulatory framework

### 2.4 Ethics approval and consent to participate

This study was approved by the Institutional Review Board of Alfaisal University, Riyadh, Saudi Arabia (IRB approval no. IRB-20161; approved 15 January 2024). All study procedures were conducted in accordance with the ethical standards of the Institutional Research Committee and the 1964 Helsinki Declaration and its later amendments. Informed consent was obtained from all participants before their participation in the interviews.

## 3 Findings

In total, 11 interviews were conducted, with data saturation reached in the 10th interview. The duration of the interviews was 45–60 min. Four key themes emerged from the analysis: data management, capability building, resource utilization, and governance (Table 1).

### 3.1 Data management

Two key subthemes demonstrate the challenges and opportunities of data management: data sharing and quality.

#### 3.1.1 Data sharing

Challenges in data sharing demonstrate the complexities healthcare systems face in effectively utilizing data for better health outcomes. A recurring concern identified by most participants was the lack of a clear framework for data governance and sharing. As one participant highlighted, “The decision of data sharing is left to the institution, if they follow the standards of SDAIA [Saudi Data and AI Authority]. But if you want to do a benchmark and comparison in collaboration with other bodies, that’s really where the picture becomes very fuzzy and unclear” (Participant 7). This sentiment was echoed by another participant, who emphasized this issue, stating, “We don’t have clear data governance as of now. Hence, regarding exchanging data—we are facing some security issues with it because of our aim to guard the privacy of the patients’ information” (Participant 5). Data sharing is also hampered by cybersecurity concerns, which can pose significant obstacles for healthcare professionals (HCPs) aiming for RWE.

In addition, the participants discussed the need for regulatory reform to support timely research initiatives. The lengthy, time-consuming regulatory approval process has been identified as a barrier to RWE progress. As one participant explained, “If you compare it with other countries, [the regulatory process] is not very efficient and very long sometimes…. Regulations should of course be there to ensure we have high-quality research. But by all means, that should not cause a delay or become an obstacle in the way of efficient research” (Participant 8).

The absence of a comprehensive data-sharing policy hinders coordination between different government entities. One participant highlighted the significance of clear coordination, stating, “We need to have very clear coordination between the different government entities…. But the data owners will not usually give their data to [these entities]…. So, we need to have clear coordination regarding this” (Participant 9).

Moreover, data ownership issues were identified as potential struggles among researchers impeding successful collaboration: “There are conflicts regarding data ownership among researchers. Some doctors claim authorship over data they only analyze, while others argue against this claim. These discussions can create friction among researchers and hinder collaboration” (Participant 6).

These findings underscore the need for collaborative efforts to advance RWE initiatives. One participant articulated, “We have a lot of difficulties when it comes to data sharing. This is actually a limiting step if you want to start any RWE/RWD collection” (Participant 4). This encapsulates the critical role of responsible data sharing in the successful initiation and implementation of RWE initiatives.

#### 3.1.2 Data quality

Many participants pointed out that incomplete or inaccurate data can severely affect research outcomes and patient care. Our thematic analysis highlighted critical areas related to data quality, including challenges in documentation and reporting, such as shallow data, missing data, inadequate details, poor documentation, and incomplete records.

For example, the participants mentioned that the data could sometimes be very shallow. “There is still no depth of data that can generate good evidence across different areas of care” (Participant 1). Furthermore, concerns were revealed regarding certain data elements as being missing or not reported accurately: “The data are not being reported in the correct manner.… A lot of things can’t be measured due to a lack of reporting” (Participant 10). This challenge of inadequately detailed reporting was echoed by another participant: “We didn’t know what the patient was on. So, the main challenge we embarked on was to start fresh and clean” (Participant 1).

Multiple participants voiced concerns about data quality in terms of accuracy, citing, for instance, examples of pregnancy records for male patients. “Unfortunately, the main issue we face regarding having high-quality data is either missing or wrong data. For example, some male patients have pregnancy-related records, and some female patients have records including diseases relating to males only” (Participant 5). Moreover, the presence of contaminated or incomplete data hindered the accurate assessment of the prevalence of severe adverse events. The issue of data quality was further emphasized, with participants acknowledging the existence of methods to address data challenges but recognizing that data quality remained a persistent problem: “Not all data are of good quality. Of course, there are methods to treat the data and overcome these challenges, but still, data quality is a problem” (Participant 9).

Additionally, the participants emphasized the importance of implementing a longitudinal data-collection strategy to enhance the depth and relevance of RWE, particularly for understanding patient outcomes over time. The implementation of such a strategy was deemed a substantial value addition to RWE initiatives. One participant emphasized, “One of the things I would love to see most is a longitudinal data strategy… It would be a big added value to RWE” (Participant 6).

### 3.2 Capability building

Participants’ perspectives revealed a pressing need for capability building as a critical step toward advancing RWE initiatives in Saudi Arabia. This could be achieved through institutionalized training and development, along with support systems for research and collaboration.

#### 3.2.1 Institutionalized training and development

Several participants emphasized the lack of trained personnel in Saudi Arabia capable of handling RWE-related tasks. Participants emphasized the need for specialized training programs to address the growing demand for RWE expertise in the Kingdom. Translating RWD into a decision requires building capability, whereas “translating RWD into a decision is something that requires building capability. We don’t have enough capable people who can do this” (Participant 4). Another participant echoed the lack of expertise in data analysis and data science, noting that this struggle poses challenges to effective research: “We were lacking in talent in the field of data science… Analyzing the data is a struggle, especially if the data size is large” (Participant 5).

Moreover, the scarcity of support, regulations, and clear guidelines for research hampers the ability of researchers and clinicians to pursue studies and implement their innovative ideas: “We lack everything except talent… [We have] researchers [and] clinicians who want to do studies and have brilliant ideas but, unfortunately, [there is a] lack of support, regulations, and clear guidelines for research” (Participant 8).

The participants called for increased efforts to train scientists and researchers in RWE. They stressed the importance of ensuring the accurate extraction and interpretation of the overwhelming amount of data, as misinterpretations can lead to erroneous conclusions: “We need to train scientists in real-world evidence… If this overwhelming number or amount of data is not used or extracted accurately, understanding their limitations, this is going to lead us to wrong interpretations” (Participant 7). Additionally, the participants emphasized the need to support newcomers navigating the field of RWE and provide them with a structured approach to facilitate their progress and develop methods for standardized data collection. By focusing on training scientists and providing structured support, Saudi Arabia can foster a skilled workforce capable of harnessing the potential of RWE and driving advancements in healthcare practices.

#### 3.2.2 Support system for research and collaboration

Participants frequently noted a pressing need for better support systems and collaborative networks to address and overcome barriers to RWE initiatives. The presence of such support systems was deemed to have the potential to encourage collaboration among researchers working in different organizations to bridge knowledge and experience gaps: “At times, when you do not have the expertise in the house, you find somebody in a different organization… So, identifying expertise outside the organization, talking to them and trying to include them within the team has been the process” (Participant 2).

However, participants also underscored the challenges posed by the lack of structure and regulations, creating uncertainty for researchers and limiting their ability to implement innovative solutions: “There are brilliant ideas, but unfortunately, due to the lack of support, regulations, and clear guidelines for research … no matter how brilliant or innovative the idea is, you won’t find the suitable kind of support” (Participant 10). Moreover, participants noted that the absence of clear data-sharing agreements has contributed to a competitive environment where researchers guard their data and refuse to share it: “A competitive environment exists among researchers, where some are reluctant to share data for fear of exposing the quality of their work. This can lead to the underutilization of existing data and resources” (Participant 10).

Another important aspect that fosters effective research and collaboration is investing in different entities, such as academic institutions, government entities, and nongovernmental organizations. This can facilitate the efficient generation of evidence and expedite informed decision-making processes: “I want different entities, whether academic institutions, government entities, or NGOs … to invest.… It’s more efficient when it comes to generating evidence and a shortcut to robust and informed decision-making” (Participant 4). Additionally, participants highlighted the importance of mentorship and the need for a system that nurtures and supports researchers, recognizing that research requires time, commitment, and guidance: “Research doesn’t happen overnight, and it doesn’t happen with a degree alone. You need a mentor, and we need to create a system that helps” (Participant 8).

Participants further emphasized the significance of fostering a culture of collaboration and support among researchers. They noted challenges related to issues of authorship and competition, highlighting the need to create an environment that encourages teamwork rather than individual recognition: “There were some issues being faced where some doctors wanted to be the first author” (Participant 6). This culture of collaboration was echoed by other participants, stressing the importance of gathering quality data by supporting one another: “We need to find a way to support each other in order to capture high-quality data … [and] remind the physicians to enter the right information into the medical records” (Participant 9).

### 3.3 Resource utilization

The participants highlighted the need to raise awareness of RWE among healthcare practitioners and patients, as its underutilization affects data quality and outcomes. Since Saudi Arabia has an advanced medical infrastructure and offers strong potential for RWE research, improvements in governance and collaboration are needed to optimize data utilization to guide investment.

#### 3.3.1 Raising awareness

Participants noted a lack of awareness among healthcare practitioners and patients regarding the significance of RWE: “The first and biggest challenge … is the lack of awareness and importance of RWE among practitioners or even patients” (Participant 11). Another participant highlighted the significance of RWD and RWE, emphasizing that RWE is growing immensely in healthcare: “Real-world evidence … is becoming a standard of practice everywhere” (Participant 7). This knowledge gap has led to the underutilization of these tools, which can, in turn, affect the quality of collected data. Raising awareness and providing education on the value of RWE were considered essential steps for improving data utilization and fostering better healthcare outcomes: “I think the first thing that we need to change is educating people better about the importance of quality of data.… If they know how important the quality of data is, it will improve” (Participant 5). Furthermore, incorporating patient perspectives is a potential avenue for enhancing the effectiveness of RWE application. One participant emphasized the importance of patient-reported outcomes and patient-generated health data: “Patient-reported outcomes and patient-generated health data will contribute a lot to healthcare outcomes.… However, the patient’s involvement still isn’t in the picture” (Participant 6).

#### 3.3.2 Investment in RWE

Saudi Arabia is seen as a promising environment for research owing to its advanced medical infrastructure and specialized expertise: “Saudi Arabia is a very fertile place for conducting research in … existing pathology and high-caliber medical centers” (Participant 8). Participants noted that effective data utilization can guide investment decisions, benefiting both public health and the economy: “If you have the kind of data sources we are talking about, you can guide these companies as to where the demand is and where they can invest” (Participant 3). Insights from multiple interviews emphasized the need for improved systems and collaborative efforts to enhance research means and data use, ultimately leading to better patient outcomes: “We have a treasure of data.… However, there isn’t clear management of this data. This indicates the need for better governance to facilitate access [to] and [the] utilization of healthcare data” (Participant 10). This type of structured approach could help to identify key areas for improvement and potential solutions, aligning with the broader objective of advancing research and healthcare quality in the region.

However, the participants expressed frustration with the biases they faced when attempting to publish Middle Eastern studies internationally. This stems from the current state of support and regulation, which undermine the quality and visibility of research from the region. A participant described the challenges faced by researchers: “When we do research and send it to magazines internationally … it’s assumed that the study is of low quality, and so it’s very challenging” (Participant 10).

### 3.4 Governance

The need for a centralized governance body was emphasized as crucial for effective utilization. Participants highlighted the importance of a clear governance structure for RWE, insisting that they need government support and the establishment of a regulatory framework.

#### 3.4.1 Regulators’ support

Government support for RWE initiatives is crucial for the sustainable growth of this field. As one participant indicated: “Who is supposed to lead RWE? Is it the SFDA [Saudi Food and Drug Authority]? Is it the Saudi Health Council?” (Participant 11). This reflects uncertainty regarding leadership in data governance and the need for coordinated effort. It was also noted that establishing a national institution to oversee data governance is essential for generating RWE for policymakers: “We need a national institution that takes this over to do two things: establishing governance for establishing these kinds of data sources [RWD] and then ensuring the quality of data sources” (Participant 3). Another participant echoed the importance of regulatory support, stating, “Without the MOH [Ministry of Health], SFDA, SDAIA, etc., it is going to be difficult” (Participant 8). Nonetheless, participants expressed optimism about the potential impact of the forthcoming national electronic medical record: “The GHO will take place.… They will announce the national electronic medical record. This could significantly enhance data integration and accessibility for research and clinical practices” (Participant 4).

#### 3.4.2 Regulatory framework

The complex nature of the RWE regulatory environment in Saudi Arabia necessitates the implementation of supportive regulations. The participants underscored the importance of unified procedures and streamlined approval processes. They also noted the absence of proper guidelines in the regulatory framework, which creates uncertainty regarding compliance with existing regulations: “If we want to move forward, we need to work in order to make the approval and regulatory process an efficient one” (Participant 8). Ethical considerations in data governance are significant and require the establishment of clear guidelines to protect privacy while promoting responsible data use.

Participants collectively stressed the need for clear regulations to support all stakeholders involved in RWE, highlighting reliance on reliable local data: “If we are talking about RWE, it is based on the concept of RWD.… There should be clear regulations to support all stakeholders. This highlights the need for a supportive regulatory framework that enhances the use of local data” (Participant 6). Participants also discussed establishing electronic platforms for managing value-based agreements. However, the lack of a centralized platform for gathering information about patients was noted as a current limitation. “Currently, we have more than 18 value-based agreements; 11 of them are already implemented and active.… The implementation of registries linked to these agreements is crucial for monitoring healthcare outcomes and improving patient care.… There is no one platform that gathers information about these patients” (Participant 1). This necessitates the establishment of interconnected electronic medical records, which are essential for gaining a comprehensive view of patient health across different institutions: “The key success element is to get all the healthcare systems’ electronic medical records connected to each other” (Participant 4).

## 4 Discussion

This is one of the first studies to explore the barriers and challenges perceived by stakeholders with regard to generating and utilization RWE related to the pharmaceutical domain in Saudi Arabia. This study’s findings reveal critical barriers, particularly regarding data quality, infrastructure, regulatory complexity, funding, and capability gaps. These challenges align with those observed globally, where a robust data infrastructure and cohesive regulatory frameworks are essential for effective RWE generation and utilization (Rudrapatna and Butte, 2020). Addressing these barriers is vital for advancing the use of RWE and RWD in the Saudi healthcare system.

The stakeholders interviewed for this study frequently reported that data quality and standardization need to be improved in several healthcare systems and research institutions. Issues such as missing data, insufficient use of coding standards, and inconsistency in documentation practices were emphasized, echoing challenges identified in the US and Europe, where RWE relies on robust, standardized data collection frameworks (Farghaly et al., 2023). This aligns with global findings, where barriers such as fragmented data systems, insufficient standardization, and underdeveloped health technology policies limit the potential for RWE generation (Babar, 2015; Farid and Baines, 2021). Additionally, the fear of data privacy and public mistrust in data sharing exacerbate these barriers, making transparent governance frameworks and stakeholder collaboration critical (Drummond et al., 2005; Rachmania and Basri, 2013). Improving these aspects is critical for achieving high-quality, reliable RWE that can inform evidence-based healthcare decisions.

A major barrier to RWD standardization identified by participants is the limited clinical applicability of current terminology standards, such as the ICD-10, which clinicians often consider too generic and cumbersome. Incorporating locally used terms can improve their usability and reduce the burden on healthcare providers (Lai et al., 2022). Effective RWD standardization requires collaboration among various stakeholders, including hospitals, data vendors, regulatory bodies, and medical product companies. Such partnerships are key to addressing diverse challenges and ensuring that RWE is reliable and actionable (Lai et al., 2022).

Although efforts to implement a national electronic medical record system in Saudi Arabia are ongoing, its timeline and feasibility remain uncertain. Participants in this study indicated the need for a national-level EHR system similar to that found in countries such as Denmark, where centralized health databases support extensive RWE generation (Makady et al., 2017). Establishing such a system will provide a strong foundation for unified data collection and analysis, enabling more robust insights into healthcare trends and outcomes.

This study also highlights regulatory challenges, with stakeholders expressing concerns over the absence of a standardized, streamlined regulatory framework. This regulatory inadequacy is a global concern as regulations often lag behind RWE capabilities (U. S. Food Drug Administration, 2018). Establishing a cohesive regulatory environment that aligns with international best practices is essential for supporting RWE and improving Saudi Arabia’s healthcare data landscape.

The findings are in alignment with Saudi Arabia’s Vision 2030, particularly its Health Sector Transformation Program. Vision 2030 aims to develop a robust, accessible, and integrated healthcare system that improves efficiency, fosters innovation, and enhances patient care through evidence-based decision-making. By identifying and addressing current obstacles in RWE generation, such as data quality, regulatory gaps, and funding limitations, this study supports Saudi Arabia’s goals of advancing public health and attracting investment in healthcare. Moreover, Vision 2030 emphasizes increased collaboration between the public and private sectors. The perspectives of stakeholders in this study regarding unified data systems and standardized processes across institutions highlight the need for such collaboration to create a cohesive, high-quality data environment. Such efforts could help position Saudi Arabia as a regional leader in data-driven healthcare innovation.

This study’s findings consistently reveal pharmaceutical companies’ reluctance to fund local RWE studies in Saudi Arabia. This barrier stems from perceptions of limited data quality, low returns on investment, and logistical challenges. Research in other contexts has shown that pharmaceutical investments tend to follow established data ecosystems, such as those in the US and EU (Zou et al., 2020). Addressing this barrier will likely require showcasing the value of Saudi data and standardizing processes across institutions to attract pharmaceutical and private-sector investments. The participants suggested creating a standardized cost structure that all institutions in Saudi Arabia would follow when providing data to external stakeholders, particularly pharmaceutical companies. This would create consistency, reduce misunderstandings, and ensure that all parties appropriately value the data. Healthcare institutions are largely responsible for implementing these changes. They must collaborate to set consistent pricing, define clear administrative processes, and establish a shared understanding of the value of the data. Such standardization would foster trust, ensure fair pricing, and attract more investment in local RWE generation efforts. Moreover, robust scientific methods are needed to generate RWE, ensuring that pertinent questions are addressed and analyzed using rigorous statistical techniques (Stey et al., 2015).

Registries and RWE initiatives face significant challenges, including difficulties in stakeholder collaboration; retaining skilled personnel such as data managers, statisticians, and epidemiologists; and securing sustainable funding, especially in the absence of government support (Stey et al., 2015; Lubbeke et al., 2019). According to the participants, a shortage of trained professionals, including data scientists and regulatory specialists, further hampers progress, highlighting the need to develop specialized talent, as emphasized in international literature (Alnofal et al., 2020). Expanding targeted training programs and fellowship opportunities for healthcare professionals in the RWE field is essential for building a sustainable talent pipeline in Saudi Arabia. Such initiatives would ensure the availability of skilled experts to drive Saudi Arabia’s RWE agenda.

The interviewed participants also emphasized the need for streamlined approval processes to support efficient RWE implementation, noting the absence of comprehensive guidelines, which creates uncertainty around compliance. Ethical considerations, particularly those related to data governance, are crucial. Stakeholders called for established protocols to safeguard privacy while enabling responsible data use, emphasizing that substantial reforms would require high-level national initiatives. Furthermore, participants were in agreement regarding the need for a dedicated national institution to oversee data governance and ensure quality control of data sources. Such an institution would serve as a central hub to coordinate efforts among the MOH, SFDA, and SDAIA, ensuring consistent progress.

Furthermore, the findings suggest that Saudi Arabia has foundational resources for advancing RWE, such as high-caliber medical institutions and available data. However, as perceived by the stakeholders, the current fragmentation and underutilization of these resources limit their potential to inform healthcare policies. Similar impediments have been observed in other studies, where concerns about the complexity, time requirements, and risks of data governance processes discourage the use of RWE for secondary purposes, thereby limiting its potential public benefits (Jones et al., 2023). Addressing these issues would align Saudi Arabia with global initiatives to improve healthcare data ecosystems and enhance the reliability of its RWE, both locally and internationally (Zou et al., 2020). In countries with well-established RWE infrastructures, such as the United Kingdom, the focus has shifted toward addressing the intricacies of data utilization, highlighting the progress Saudi Arabia could make by adopting similar frameworks (Farghaly et al., 2023).

Implementing such changes requires a coordinated effort. This includes leadership from high-level decision-makers, policymakers, and stakeholders who can guide the standardization process. Participants suggested workshops, symposiums, and collaborative meetings as platforms on which institutions could align their approaches and unified systems. There is also a need to increase awareness among institutions regarding the intrinsic and economic value of healthcare data. Educating leaders and administrative teams can help ensure that data are treated as valuable assets, potentially leading to standardized approaches that maximize their use.

The involvement of health system stakeholders (governmental, semi-governmental, and private) is vital for advancing RWE and healthcare research in Saudi Arabia. These stakeholders drive healthcare policies, ensure the safety and efficacy of pharmaceuticals, and support clinical trials and research. Collaboration among stakeholders creates a robust ecosystem to support evidence-based healthcare innovations and ensures better outcomes for the Saudi population.

To overcome the identified barriers to generating RWE, Saudi Arabia can implement strategies proven effective by nations with advanced RWE ecosystems. Denmark and Sweden, for instance, have implemented centralized national health databases that enable comprehensive, standardized data collection across healthcare institutions (Blacketer et al., 2025). Similarly, the United Kingdom’s NHS electronic platform offers robust data governance frameworks and clear protocols for ethical access and usage—models that Saudi Arabia could emulate by establishing a national data governance body in collaboration with key stakeholders. Accelerating the development of a unified EHR system would further support integration and enhance data quality for RWE (Unsworth et al., 2021; Christie, 2022).

Other international practices can offer guidance regarding stakeholder engagement, workforce development, and funding. Singapore’s success with public–private partnerships demonstrates the value of standardized cost structures and regulatory incentives to attract pharmaceutical investment in RWE initiatives (Ballantyne et al., 2022). The US addresses workforce shortages by offering training programs in regulatory science and RWE analytics, and Saudi Arabia could replicate this through institutions such as the King Abdullah International Medical Research Center and King Faisal Specialist Hospital and Research Centre (Dagenais et al., 2022). Additionally, regular multi-stakeholder workshops and national summits, as seen in Canada and Australia, could facilitate the alignment of data standards, governance, and research priorities. Adapting these global best practices could help Saudi Arabia build a strong, sustainable RWE ecosystem that aligns with Vision 2030 and supports data-driven healthcare innovation (Zhang et al., 2022).

Moreover, countries such as the United Kingdom, Denmark, and Singapore have demonstrated that successful RWE ecosystems depend not only on standardized national data infrastructure and regulatory clarity but also on strategic innovation, particularly the integration of artificial intelligence (AI) in data processing and analytics. AI has shown substantial potential for improving the efficiency, scalability, and accuracy of RWD analysis, particularly through natural language processing, predictive modeling, and automated data harmonization (Rudrapatna and Butte, 2020; Zhang et al., 2022). In nations with advanced RWE systems, AI is increasingly used to extract insights from unstructured clinical notes, detect patterns in longitudinal datasets, and streamline patient cohort identification for observational studies (Rose and Chen, 2024).

With its ambitious Vision 2030 and strong national institutions such as the SDAIA, Saudi Arabia is well-positioned to leverage AI to enhance RWE capabilities. Incorporating AI-driven tools into national registries and EHR systems can dramatically improve data standardization and quality, reduce manual errors, and accelerate the generation of evidence. However, such transformation will require investment in workforce upskilling, ethical data governance, and AI-specific infrastructure, such as high-performance computing environments and interoperable data lakes.

By adapting international best practices—such as Denmark’s centralized health databases, the United Kingdom’s NHS Digital, and Singapore’s public–private RWE partnerships—Saudi Arabia can build a future-ready RWE ecosystem. Embracing AI as a strategic enabler in this journey will not only improve operational efficiency but also unlock new possibilities in data-driven, patient-centered healthcare innovation (Christie, 2022; Zaabi and Padela, 2024). In doing so, Saudi Arabia can position itself as a regional leader in real-world research and ensure that healthcare decisions are informed by timely, robust, and actionable evidence.

The diversity of this study’s participants strengthened the findings, making them applicable to different aspects of the Saudi healthcare ecosystem. Moreover, the consistency of the interview process ensured that responses were directly comparable, providing a clear and structured understanding of the barriers to RWE across institutions. The consistent questioning enhances the study’s reliability, as it captures a range of perspectives on the same issues, ultimately supporting more accurate and cohesive analysis. Nevertheless, this study has some limitations that should be acknowledged. First, the sample size was limited to 11 stakeholders, which, although sufficient to reach data saturation, may not have captured a full diversity of perspectives across all regions and institutions in Saudi Arabia. That said, the findings of this research are not intended to be statistically generalizable. Instead, they offer rich contextual insights that can be transferred to similar settings and theoretical propositions. Second, as a qualitative study, the findings are inherently interpretive and context-specific, limiting their generalizability beyond the interviewed population. Additionally, there may have been response bias, as participants could have provided socially desirable answers due to their affiliations or professional roles. Finally, the reliance on self-reported data through interviews means that some insights may reflect perceptions more than verifiable organizational practices. Future research could incorporate quantitative methods or mixed approaches and expand the stakeholder pool to include frontline clinicians and more private-sector representatives for a more holistic understanding.

## 5 Conclusion

This study highlights the barriers stakeholders perceive as impeding RWE generation and utilization in Saudi Arabia for use in pharmaceutical research and decision-making. Despite such challenges, Saudi Arabia’s infrastructure and Vision 2030 objectives provide a strong foundation for the advancement of RWE. Adopting international best practices in data governance and management, AI integration, and stakeholder collaboration could drive system-wide improvements. Investing in workforce development and public–private partnerships is crucial for system sustainability. A unified national data-governance body is essential for ensuring consistency and transparency. These findings highlight actionable steps that could strengthen Saudi Arabia’s position as a regional leader in RWE. Adopting such strategic initiatives to enhance the RWE ecosystem could ultimately support evidence-based decision-making and improve population health outcomes.

## Data Availability

The original contributions presented in the study are included in the article/Supplementary Material, further inquiries can be directed to the corresponding author.
