# Supplementary material for: Navigating barriers to real-world evidence utilization for drug regulatory affairs and market access in Saudi Arabia
Source: Front Pharmacol. 2025 Dec 2;16:1712147. doi: 10.3389/fphar.2025.1712147 (PMC12705390; doi:10.3389/fphar.2025.1712147)
Supplement: Supplementary file 1 [file DataSheet2.docx]

**Appendix 2. Interview Topic Guide Questions Development, Validation, and Piloting Process**

1. Description of the expert panel review process (3 experts in qualitative research and RWE)
2. Details of the pilot interview conducted with one stakeholder (not included in the study findings on interviewed participants)
3. Modifications made based on pilot feedback (e.g., clarifying terminology, adjusting probe questions)
4. Final approval process by the research team

The expanded Appendix 1 now includes:

**DEVELOPMENT AND VALIDATION PROCESS:**The interview guide was developed through a systematic, multi-stage process:

**1. Initial Development (September 2024):** - Research team (3 members) conducted literature review of RWE barriers globally
 - Identified key domains: data management, capabilities, resources, governance
 - Drafted initial question set with 3 core questions and 12 probes

**2. Expert Review and Validation (October 2024):**
 - Expert panel consisting of:
 • 2 qualitative research methodologists from Alfaisal University
 • 1 RWE subject matter expert from pharmaceutical industry
 - Panel reviewed for: content validity, clarity, neutrality, and comprehensiveness
 - Revisions made based on expert feedback:
 • Simplified technical terminology
 • Added probes for data quality and sharing
 • Refined questions to reduce researcher bias

**3. Pilot Testing (October 2024):**
 - Conducted pilot interview with 1 senior stakeholder (regulatory background)
 - Pilot participant NOT included in final study sample
 - Duration: 52 minutes
 - Modifications based on pilot:
 • Clarified meaning of "real-world evidence" at interview start
 • Adjusted probe questions for better flow
 • Added examples to help participants understand context
 • Confirmed 45-60 minute timeframe was appropriate

**4. Final Approval:**
 - Research team reviewed all modifications
 - Final version approved by all co-investigators
 - Implemented for data collection starting November 2024
