# Supplementary material for: Navigating barriers to real-world evidence utilization for drug regulatory affairs and market access in Saudi Arabia
Source: Front Pharmacol. 2025 Dec 2;16:1712147. doi: 10.3389/fphar.2025.1712147 (PMC12705390; doi:10.3389/fphar.2025.1712147)
Supplement: Supplementary file 2 [file DataSheet1.docx]

**Appendix I. Interview Topic Guide**

The aim of this study was to explore the challenges faced by pharmaceutical stakeholders in the Kingdom of Saudi Arabia (KSA) regarding the generation and investment in real-world evidence (RWE) studies. Although RWE has long been recognized in the global literature, its local adoption remains limited. The current terminology increasingly refers to real-world data (RWD) as a tool for assessing long-term efficacy of already marketed medications. Despite the growing importance of these large-scale studies, investment and engagement from pharmaceutical companies in KSA appear suboptimal.

This interview guide was developed to investigate the potential limitations hindering the advancement of RWE in the region, whether they relate to data availability, regulatory frameworks, lack of enablers, or other systemic factors.

1. Can you describe your experience with generating or using real-world evidence (RWE)?
2. In your opinion, what are the primary barriers faced by stakeholders—including yourself—in generating RWE in KSA?
3. What interventions or strategies do you believe could improve the uptake, implementation, and overall maturity of RWE in KSA?

The followings are generic probing and prompting questions that were used in the interviews with participants:

1. Could you explain that further?
2. Could you please give me an example?
3. What do you mean by that?
4. Can you tell me more about that?
5. Can you explain that in a different way?
6. Is there anything else you would like to say about this?
